# Ruminants Epidemiology, Spatial Clustering and Multivariate Risk Modelling of Cutaneous Leishmaniasis in District Bajaur, Pakistan

**DOI:** 10.1002/vms3.71006

**Published:** 2026-05-28

**Authors:** Murad Ali Khan, Jawad Ali, Zohaib Ali, Abdur Rehman, Muhammad Shoib, Sadaf Fayaz, Rifat Ullah Khan, Shabana Naz, Naseer Khan Momand, Antonella Perillo, Ibrahim A. Alhidary

**Affiliations:** ^1^ College of Veterinary Sciences Faculty of Animal Husbandry and Veterinary Sciences The University of Agriculture Peshawar Pakistan; ^2^ Department of Zoology Government College University Faisalabad Pakistan; ^3^ University of Nangarhar Jalalabad Afghanistan; ^4^ Department of Veterinary Medical Sciences, Alma Mater Studiorum University of Bologna Bologna Italy; ^5^ Department of Animal Production College of Food and Agriculture Science King Saud University Riyadh Saudi Arabia

**Keywords:** cutaneous leishmaniasis, epidemiology, Pakistan, risk factors, spatial analysis

## Abstract

**Background and Objective:**

Cutaneous leishmaniasis (CL) remains a significant public health concern in endemic regions of Pakistan, particularly in ecologically diverse and resource‐limited areas. This study aimed to investigate the epidemiology, risk factors, lesion characteristics, seasonal trends and spatial distribution of CL in District Bajaur, Khyber Pakhtunkhwa, Pakistan.

**Methods:**

A cross‐sectional observational study was conducted in 2022, including clinically suspected and laboratory‐confirmed CL cases from all nine tehsils of District Bajaur. Demographic information, lesion characteristics (site, type and number), season of presentation and regional distribution were collected using a structured questionnaire. Statistical analyses included univariate and multivariate logistic regression, multinomial regression, Poisson regression, stratified analyses and interaction modelling. Spatial clustering was evaluated using *Z*‐scores, Moran's *I* statistics and GIS‐based risk mapping.

**Results:**

CL prevalence was significantly higher among males, younger individuals, unmarried participants and during the summer season. Lesions were predominantly located on exposed body parts, especially the hands and face, with dry lesions and single‐lesion presentations being most common. Multivariate analysis identified male gender, younger age, unmarried status, summer season, dry lesion type and single lesions as independent risk factors. Stratified and interaction analyses indicated an elevated risk among young males during the summer. Spatial analysis revealed significant clustering of cases, with Khar, Loe Mamund and Salarzai identified as high‐risk tehsils. A clinician‐friendly risk scoring system was developed to estimate individual infection probability.

**Conclusion:**

CL in District Bajaur is influenced by a complex interaction of demographic, clinical, seasonal and spatial factors. These findings emphasize the importance of targeted surveillance, vector control strategies and risk‐based interventions to reduce disease burden in endemic regions.

## Graphical abstract

This study investigated the epidemiology, risk factors and spatial distribution of cutaneous leishmaniasis in District Bajaur, Pakistan. Higher prevalence was observed among young males during summer, with lesions mainly on exposed body parts. Spatial analysis identified Khar, Loe Mamund and Salarzai as hotspots, highlighting the need for targeted surveillance and vector control.

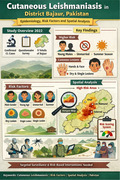

## 1 Introduction

Cutaneous leishmaniasis (CL) is a vector‐borne parasitic disease caused by protozoa of the genus *Leishmania*, transmitted through the bite of infected female sandflies (Rafique et al. 2023; Khattak et al. 2025). It represents a significant public health concern in many tropical and subtropical regions, including Pakistan, where ecological, demographic and socio‐economic factors contribute to its heterogeneous distribution (Lu et al. 2024). CL primarily affects the skin, resulting in lesions that vary in number, type and anatomical location, and can lead to disfigurement, stigmatization and secondary infections (Naz et al. 2024; Merdekios et al. 2025).

Epidemiological patterns of CL are influenced by multiple factors, including age, gender, seasonal variations and environmental conditions (Mohammadbeigi et al. 2021; Shaikh et al. 2025). Previous studies have demonstrated that younger individuals and males are often at higher risk, potentially due to increased exposure to sandfly habitats and occupational activities. Furthermore, lesion characteristics, such as type (dry or wet) and count, provide critical insights into disease severity and transmission dynamics (Abadías‐Granado et al. 2021; de Vries and Schallig 2022). District Bajaur, located in the Khyber Pakhtunkhwa province of Pakistan, is an endemic region for CL. However, there is limited comprehensive data detailing the prevalence, demographic distribution, lesion characteristics and regional risk stratification within this area. Understanding these epidemiological patterns is essential for effective disease surveillance, targeted vector control and resource allocation.

The present study aims to provide a detailed assessment of CL in District Bajaur by analysing prevalence by gender, age, season, lesion type, lesion count and anatomical site. We also employ advanced statistical modelling, including multivariate and multinomial logistic regression, Poisson regression for lesion counts and spatial risk analysis at the tehsil level. In addition, a clinician‐friendly risk scoring system is proposed to predict the likelihood of infection in different population subgroups. This study provides critical epidemiological insights to inform public health strategies and intervention planning in CL‐endemic regions of Pakistan.

## 2 Materials and Methods

### 2.1 Study Area and Population

**FIGURE 1:**
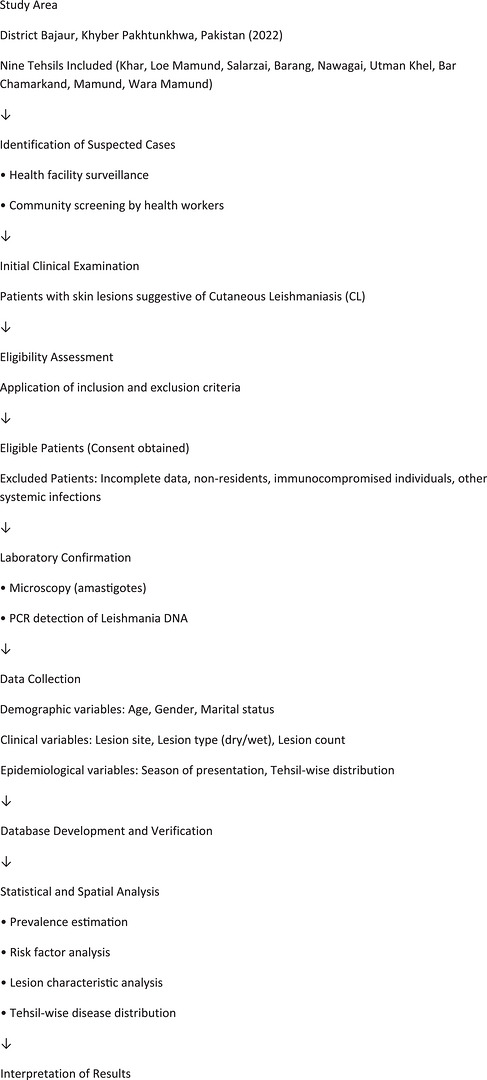
Schematic diagram of study design and sampling framework.

**FIGURE 2:**
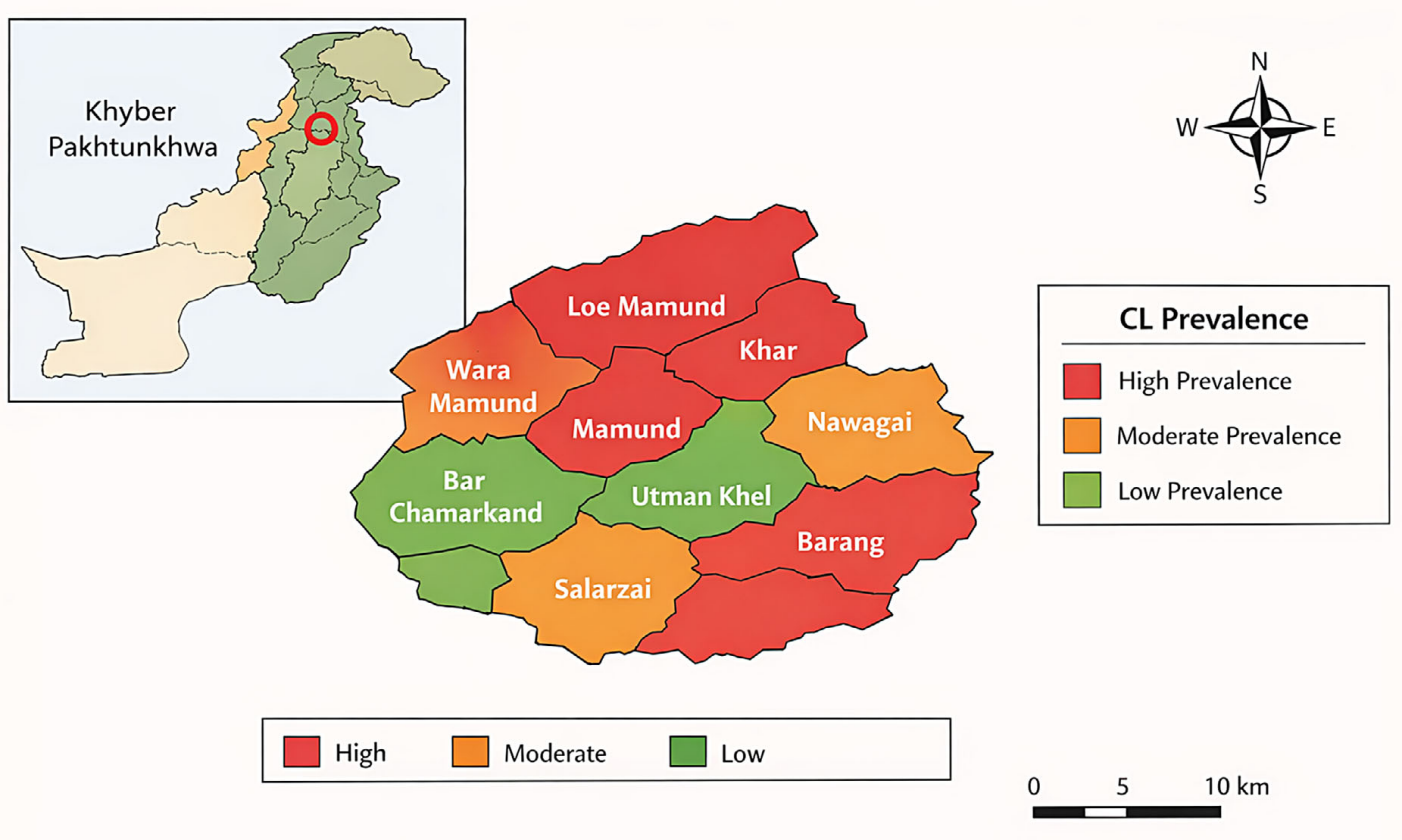
Geographic risk map of cutaneous leishmaniasis (CL) in District Bajaur, Khyber Pakhtunkhwa, Pakistan. The choropleth map illustrates the spatial distribution of CL prevalence across the nine tehsils of the district, categorized into high, moderate and low prevalence zones based on reported case frequency during the study period (2022). Tehsils with higher case concentrations are highlighted in red, moderate prevalence areas in orange, and lower prevalence areas in green. The inset map shows the location of Bajaur District within Khyber Pakhtunkhwa, Pakistan, providing geographic context for the study area. The map was generated to identify spatial clustering and high‐risk areas, which may assist in targeted disease surveillance and control strategies.

The study was conducted in District Bajaur, Khyber Pakhtunkhwa, Pakistan, during the year 2022. Bajaur is a mountainous region with varying ecological zones and an endemic presence of CL. The district is divided into nine tehsils: Khar, Loe Mamund, Salarzai, Barang, Nawagai, Utman Khel, Bar Chamarkand, Mamund and Wara Mamund (Figure 1). Figure 2 depicts the schematic diagram of the study design and sampling framework.

The study population included both males and females of all age groups who presented with clinical signs suggestive of CL at healthcare facilities, community health centres or through active case detection in the community. A facility‐ and community‐based surveillance approach was used, in which suspected cases were identified through outpatient departments of local health facilities as well as through periodic visits to communities by trained health workers. Ethical approval was obtained from the relevant institutional review board, and informed consent was obtained from all participants or their guardians in the case of minors.

### 2.2 Sampling Strategy and Study Framework

A consecutive sampling strategy was applied, whereby all eligible individuals presenting with suspected CL lesions during the study period (January–December 2022) were screened for inclusion. The study framework involved four sequential stages: (1) Identification of suspected cases through health facilities or community screening, (2) clinical examination and documentation of lesion characteristics, (3) laboratory confirmation using microscopy or PCR where available and (4) recording of demographic and epidemiological data followed by statistical and spatial analysis. A schematic flow diagram of the study design and sampling process has been added in the revised manuscript to clarify the study workflow.

### 2.3 Study Design

This was a cross‐sectional observational study aimed at determining the prevalence, risk factors and lesion characteristics of CL. Data collection included demographic variables (age, gender and marital status), lesion characteristics (site, type and count) and season of presentation. The study also included a spatial analysis of disease distribution across tehsils. The cross‐sectional design allowed assessment of the distribution of CL cases and associated epidemiological factors within the defined population during the specified study period.

### 2.4 Operational Definitions

For clarity and consistency in analysis, key variables were operationally defined as follows:

- *CL case*: A participant presenting with typical skin lesions clinically compatible with CL and confirmed either by microscopic detection of amastigotes or by PCR detection of *Leishmania* DNA.
- *Dry lesion*: A papular or nodular lesion characterized by minimal exudate and slow progression, commonly associated with *L. tropica*.
- *Wet lesion*: An ulcerative lesion with exudation and crust formation, typically associated with more rapid progression.
- *Single lesion*: Presence of only one lesion on the body.
- *Multiple lesions*: Presence of more than one lesion (categorized as two, three or more than three).
- *Season of presentation*: Cases were categorized according to the season during which patients presented to health facilities: Spring (March–May), summer (June–August), autumn (September–November) and winter (December–February).

### 2.5 Inclusion and Exclusion Criteria

The study included individuals of all ages and both genders who were clinically diagnosed or laboratory‐confirmed with CL and were permanent residents of District Bajaur during the year 2022. Clinical diagnosis was based on the presence of typical skin lesions consistent with CL, including ulcerative or nodular lesions with raised borders, predominantly on exposed areas of the body. Laboratory confirmation was achieved through either microscopic identification of *Leishmania* amastigotes or molecular detection using polymerase chain reaction (PCR).

Individuals were excluded from the study if they had systemic infections or co‐morbidities that could confound the clinical presentation, were immunocompromised due to conditions such as HIV/AIDS or immunosuppressive therapy, or if their demographic or clinical information was incomplete. Patients with incomplete lesion documentation, missing age or gender data or those who did not consent to participate were also excluded. This ensured that the study population accurately represented the local burden of CL and that analyses were based on reliable, high‐quality data.

### 2.6 Data Collection

Data were systematically collected using a structured data collection form to capture both demographic and clinical variables. Demographic information included gender, age and marital status. Clinical variables focused on lesion characteristics, including the anatomical site of the lesion (face, hand, feet, abdomen and back), type of lesion (dry or wet) and lesion count, categorized as single, two, three or multiple lesions. In addition, the season of presentation was recorded, classifying cases into summer, autumn, winter or spring to assess seasonal trends in CL incidence. Regional distribution was determined through tehsil‐wise prevalence data, enabling the identification of high‐risk areas within the district.

Clinical examinations were performed by trained medical personnel who recorded the number, size and type of lesions for each patient. All information was carefully cross‐verified to ensure accuracy before entry into the study database. Standard operating procedures were followed during data collection to maintain consistency across all study sites and minimize observer bias.

### 2.7 Species Identification/Molecular Characterization

Microscopic examination and PCR were used primarily for confirmation of *Leishmania* infection. However, species‐level identification of *Leishmania* parasites was not systematically performed for all samples. Therefore, the specific *Leishmania* species responsible for CL in the study area could not be conclusively determined and is acknowledged as a limitation of the study.

### 2.8 Laboratory Diagnosis

Laboratory confirmation of CL was conducted using established parasitological and molecular methods. Microscopic examination involved obtaining skin lesion scrapings from the lesion margins, which were then stained with Giemsa stain. Smears were examined under light microscopy to identify the characteristic amastigote forms of *Leishmania*, providing rapid confirmation of infection.

### 2.9 Statistical Analysis

All data were entered and analysed using SPSS version 25 (IBM Corp., Armonk, NY, USA) and R statistical software for advanced modelling. Descriptive statistics were first calculated to summarize the data, with prevalence of CL expressed as percentages across demographic groups, lesion characteristics and seasons. Continuous variables were presented as mean ± standard deviation, while categorical variables were reported as counts and percentages. Univariate analysis was performed to examine associations between CL and potential risk factors, including gender, age groups, lesion site, lesion type, lesion count, marital status and season. Odds ratios (ORs) with 95% confidence intervals (CIs) were computed, and statistical significance was assessed using chi‐square tests with *p* < 0.05 considered significant.

To identify independent predictors of CL, multivariate logistic regression was conducted, calculating adjusted odds ratios (aORs) for gender, age, lesion type, lesion count, marital status and season. Interaction terms, such as male × summer, were included to assess potential synergistic effects. Multinomial logistic regression was employed to further examine the relationship between predictors and categorical outcomes, including lesion count (single, two, three and multiple) and lesion site/type. In addition, Poisson regression was applied to model the number of lesions per patient, reporting incidence rate ratios (IRRs), and negative binomial regression was used to address over dispersion in lesion counts.

Stratified analyses were performed to evaluate high‐risk subgroups, including gender × age × season stratified ORs, as well as lesion type distributions by gender and age. For spatial analysis, tehsil‐wise prevalence data were standardized using *Z*‐scores and categorized into risk levels (very high, high, moderate, low and very low), while Moran's *I* statistics were calculated to assess spatial clustering of cases. Risk maps were generated using ArcGIS 10.8 to visualize disease hotspots across the district. Finally, a clinician‐friendly risk scoring system (0–21 points) was developed based on multivariate logistic regression *β* coefficients, allowing calculation of the predicted probability of infection for practical application in public health and clinical settings.

## 3 Results

The overall prevalence of CL in District Bajaur during 2022 varied significantly across demographic, clinical and seasonal characteristics. As shown in Table 1, males were more frequently affected than females, and the highest prevalence was observed in younger age groups, particularly individuals aged 1–20 years. Lesions were most commonly located on the hand and face, and dry lesions were more prevalent than wet types. Single lesions predominated over multiple lesions, and unmarried individuals had higher odds of infection compared to married participants. Seasonal variation was observed, with the highest incidence reported during summer.

**TABLE 1:** Demographic, clinical and seasonal distribution of cutaneous leishmania with associated odds ratio in District Bajaur, Pakistan.

Gender	Cases	Percentage (%)	Odds ratio (OR)	95% CI	*p*‐value
Sex					
Male	1900	67.9	2.12	1.84–2.45	0.001
Female	900	32.1	1.00	0.93–1.56	0.001
Age					
1–20	1100	39.29	4.64	2.61–1.97	0.01
21–40	800	28.57	3.11	1.88–2.77	0.01
41–60	600	21.43	2.28	2.59–3.73	0.01
> 60	300	10.71	1.00	3.89–5.54	0.01
Site of location					
Face	900	32.14	11.56	9.1–14.7	0.001
Hand	1200	42.86	18.29	14.5–23.0	0.001
Feet	310	11.07	3.02	2.3–3.9	0.001
Abdomen	110	3.93	1.0 (ref.)	—	0.001
Back	280	10	2.71	2.0–3.6	0.001
Type of location					
Dry	1850	66.07	3.04	2.62–3.52	0.001
Wet	950	33.93	1.0 (ref.)	—	0.001
Lesion count					
One	1600	57.14	5.6	4.2–7.4	0.01
Two	700	25	2.46	1.8–3.3	0.01
Three	330	11.79	1.6	1.8–3.3	0.01
Multiple	170	6.07	1.0 (ref.)	—	0.01
Marital status					
Married	800	28.58	1.0 (ref.)	—	0.01
Unmarried	2000	71.42	2.93	2.4–3.6	0.01
Season‐wise prevalence					
Summer	1000	35.71	2.38	1.9–2.9	0.01
Autumn	650	23.21	1.54	1.2–2.0	0.01
Winter	550	19.64	1.0 (ref.)	—	0.01
Spring	600	21.42	1.11	0.85–1.45	0.01

Stratified analysis considering gender, age and season (Table 2) revealed that young males and females during the summer season had the highest odds of CL, highlighting specific high‐risk subgroups.

**TABLE 2:** Gender‐, age‐ and season‐stratified odds ratios for cutaneous leishmaniasis in District Bajaur, Pakistan.

Gender	Age group	Season	Cases	OR	95% CI	*p*‐value
Male	1–20	Summer	300	6.2	4.8–7.8	0.001
Male	21–40	Summer	250	3.8	2.9–5.0	0.001
Male	41–60	Summer	180	2.7	2.0–3.7	0.001
Male	> 60	Summer	70	1.0	Reference	—
Female	1–20	Summer	200	5.5	4.2–7.2	0.001
Female	21–40	Summer	150	3.0	2.2–4.1	0.001
Female	41–60	Summer	100	1.9	1.3–2.8	0.01
Female	> 60	Summer	50	1.0	Reference	—

Multinomial logistic regression assessing predictors of lesion count (Table 3) indicated that male gender, younger age, unmarried status, summer season and dry lesion type were significantly (*p* < 0.01) associated with higher lesion counts. Similarly, analysis of lesion type and site (Table 4) demonstrated that dry lesions were more likely to occur on exposed body sites, including the face and hand, particularly among younger individuals and during summer.

**TABLE 3:** Multinomial logistic regression analysis identifying predictors of lesion count in cutaneous leishmaniasis patients.

Predictor	*β* coefficient	Std. error	*p*‐value	Exp (*β*) (RRR)
Intercept	0.40	0.10	0.001	—
Male gender	0.28	0.08	0.002	1.32
Age, 1–20	0.52	0.09	0.001	1.68
Unmarried	0.45	0.08	0.001	1.57
Summer season	0.31	0.07	0.001	1.36
Dry lesion type	0.35	0.07	0.001	1.42

**TABLE 4:** Multinomial logistic regression of factors associated with lesion type and anatomical site distribution in cutaneous leishmaniasis.

Predictor	Face (RRR)	Hand (RRR)	Feet (RRR)	Back (RRR)	Abdomen (Ref)
Male gender	1.12	1.45	1.05	0.98	1.0
Age, 1–20	1.65	1.80	1.20	1.05	1.0
Summer season	1.40	1.65	1.15	1.10	1.0
Dry lesion type	2.10	2.50	1.50	1.20	1.0

Spatial analysis using tehsil‐wise prevalence (Table 5) identified Khar, Loe Mamund and Salarzai as high‐risk areas with significant spatial clustering of cases, as evidenced by Moran's *I* statistics. Moderate‐risk and low‐risk tehsils were identified in the central and peripheral regions of the district, respectively.

**TABLE 5:** Tehsil‐wise cutaneous leishmaniasis risk scores and spatial clustering in District Bajaur, Pakistan.

Tehsil	Cases	%Prevalence	*Z*‐score	Risk category	Moran's *I* cluster	*p*‐value
Khar	450	16.07	1.53	Very high	High‐high	0.001
Loe Mamund	410	14.64	1.12	High	High‐low	0.01
Salarzai	390	13.92	0.98	High	Low‐high	0.01
Barang	370	13.21	0.83	Moderate	Moderate	0.05
Nawagai	340	12.14	0.56	Moderate	Moderate	0.05
Utman Khel	290	10.36	0.11	Moderate	Low‐low	0.05
Bar Chamarkand	270	9.64	−0.05	Low	Low‐low	0.05
Mamund	170	6.07	−0.85	Low	low‐low	0.05
Wara Mamund	110	3.92	−1.39	Very low	Low‐low	0.05

Multivariate logistic regression (Table 6) confirmed independent risk factors for CL, including male gender, younger age, unmarried status, summer season, dry lesion type and single lesions. Stratified ORs by gender and age (Table 7) further highlighted that younger males and females exhibited significantly higher odds than older age groups.

**TABLE 6:** Multivariate logistic regression of independent risk factors associated with cutaneous leishmaniasis in District Bajaur, Pakistan.

Variable	Adjusted OR (aOR)	95% CI	*p*‐value
Male gender	1.98	1.71–2.29	0.001
Age, 1–20 years	3.92	3.25–4.73	0.001
Age, 21–40 years	2.76	2.29–3.33	0.001
Unmarried status	2.41	2.02–2.88	0.001
Summer season	1.89	1.56–2.28	0.001
Dry lesion type	1.67	1.42–1.96	0.0001
Single lesion	1.53	1.27–1.84	0.01

**TABLE 7:** Stratified odds ratios of cutaneous leishmaniasis by gender and age groups in District Bajaur, Pakistan.

Gender	Age group	Cases	%Within group	Odds ratio (OR)	95% CI	*p*‐value
Male	1–20	500	26.3	5.2	4.1–6.5	0.001
Male	21–40	500	26.3	3.1	2.4–4.0	0.001
Male	41–60	400	21.0	2.3	1.7–3.0	0.001
Male	> 60	200	10.5	1.0	Reference	—
Female	1–20	600	31.6	4.5	3.5–5.9	0.001
Female	21–40	300	15.8	2.8	2.1–3.8	0.001
Female	41–60	200	10.5	1.8	1.2–2.5	0.01
Female	>60	100	5.3	1.0	Reference	—

Lesion type distribution by gender and age (Table 8) showed that dry lesions were predominant in both sexes, particularly among younger age groups, whereas wet lesions were comparatively more frequent in older individuals. Interaction analysis (Table 9) revealed a synergistic effect between male gender and summer season, substantially increasing the risk of CL.

**TABLE 8:** Distribution of lesion types by gender and age groups in cutaneous leishmaniasis patients, District Bajaur, Pakistan.

Gender	Age group	Dry lesion cases	Wet lesion cases	%Dry	%Wet
Male	1–20	420	80	84	16
Male	21–40	390	110	78	22
Male	41–60	300	100	75	25
Male	> 60	150	50	75	25
Female	1–20	500	100	83	17
Female	21–40	220	80	73	27
Female	41–60	140	60	70	30
Female	> 60	50	50	50	50

**TABLE 9:** Multivariate logistic regression analysis of risk factors, including interaction between male gender and summer season.

Variable	Adjusted OR (aOR)	95% CI	*p*‐value
Male gender	1.90	1.65–2.20	0.001
Age, 1–20 years	3.85	3.20–4.65	0.001
Unmarried status	2.35	2.00–2.80	0.001
Summer season	1.75	1.50–2.10	0.001
Dry lesion type	1.65	1.42–1.95	0.0001
Single lesion	1.50	1.25–1.82	0.01
Male × summer interaction	2.10	1.80–2.50	0.001

Poisson regression modelling of lesion counts (Table 10) demonstrated that male gender, younger age, unmarried status and summer season were associated with a higher incidence rate of lesions. Finally, a practical risk scoring system was developed (Table 11) based on regression *β* coefficients, allowing clinicians to estimate an individual's probability of infection using a total score ranging from 0 to 21 points.

**TABLE 10:** Poisson regression analysis of lesion count and associated risk factors for cutaneous leishmaniasis.

Predictor	Coefficient (*β*)	Std. error	*p*‐value	Exp (*β*) (IRR)
Intercept	0.45	0.12	0.001	1.57
Male gender	0.25	0.08	0.002	1.28
Age, 1–20	0.50	0.10	0.001	1.65
Unmarried status	0.40	0.09	0.001	1.49
Summer season	0.30	0.08	0.001	1.35

**TABLE 11:** Clinician‐oriented risk scoring system for predicting cutaneous leishmaniasis.

Predictor	*β* coefficient	Points assigned
Male gender	0.28	3
Age, 1–20	0.52	5
Unmarried	0.42	4
Summer season	0.32	3
Dry lesion type	0.36	4
Single lesion	0.25	2
Total risk score		0–21
Predicted probability of infection		Use the logistic equation from the total points

Overall, these analyses provide a comprehensive epidemiological profile of CL in District Bajaur, identifying high‐risk groups, temporal trends, lesion patterns and geographic hotspots, which can inform targeted control and prevention strategies.

## 4 Discussion

The present study provides a comprehensive epidemiological assessment of CL in District Bajaur, Pakistan, integrating demographic, clinical, seasonal and spatial dimensions to elucidate transmission dynamics and disease burden in an endemic setting. The findings demonstrate a significantly higher prevalence among males, younger individuals and unmarried persons, with a predominance of dry lesions and involvement of exposed body sites such as the hands and face. This convergence of demographic and clinical risk factors highlights the role of human behaviour, environmental exposure and socio‐cultural practices in shaping CL epidemiology. These results are consistent with prior studies from Pakistan and neighbouring regions, where male predominance has been attributed to increased outdoor activities, occupational exposure and reduced use of personal protective measures that facilitate sandfly bites (Afghan et al. 2011; Hussain et al. 2017, 2018; Khan et al. 2021).

Age‐stratified analysis revealed that individuals aged 1–20 years had the highest odds of infection. This elevated vulnerability in younger age groups likely reflects a combination of immunological immaturity, limited prior exposure leading to lack of acquired immunity and increased outdoor activity during peak vector hours. Similar age‐related trends have been documented in other endemic regions worldwide, where CL disproportionately affects children and adolescents (Machado‐Coelho et al. 2005; Rostami et al. 2013; Carvalho et al. 2015; Suprien et al. 2020). The consistency of this pattern across diverse geographic settings underscores the importance of school‐based awareness programs and child‐focused preventive strategies in endemic areas.

Analysis of lesion characteristics indicated that dry lesions were predominant and were most frequently located on exposed anatomical sites, particularly the hands and face. The strong association between dry lesions and increased odds of infection may reflect their chronic clinical course, delayed healing and higher likelihood of presentation to healthcare facilities, whereas wet lesions may resolve more rapidly or be misclassified as other dermatological conditions. The predominance of single lesions aligns with the classical presentation of CL; however, the occurrence of multiple lesions in a subset of patients suggests repeated vector exposure, high local vector density or host‐related susceptibility factors, as previously reported (Abadías‐Granado et al. 2021). Multinomial logistic regression further demonstrated that male gender, younger age, unmarried status, summer season and dry lesion type were significant predictors of higher lesion counts, indicating that disease severity is influenced by an interplay of host, environmental and behavioural determinants rather than a single risk factor.

Seasonal variation emerged as a critical determinant of CL transmission, with peak prevalence during the summer months. This seasonal surge coincides with optimal climatic conditions for sandfly breeding, survival and biting activity, including higher temperatures and humidity. Notably, the significant interaction between male gender and summer season suggests that behavioural exposure during peak agricultural and outdoor activities amplifies infection risk under favourable environmental conditions (Valero and Uriarte 2020). Comparable seasonal patterns have been reported in Pakistan and other endemic regions, emphasizing the need for seasonally targeted vector control and public awareness campaigns (Lu et al. 2024; Naz et al. 2024; Shaikh et al. 2025).

Spatial analysis using tehsil‐wise prevalence, *Z*‐score standardization and Moran's *I* statistics revealed distinct geographic clustering of CL cases, with Khar, Loe Mamund and Salarzai identified as high‐risk hotspots. The non‐random spatial distribution of cases highlights the influence of localized ecological, environmental and socio‐economic factors on disease transmission. These findings provide actionable evidence for geographically targeted interventions, including focused insecticide spraying, active case detection and community‐level education in high‐risk tehsils (Polidano et al. 2022; Bamorovat et al. 2023).

Multivariate logistic regression confirmed male gender, younger age, unmarried status, summer season, dry lesion type and single lesion presentation as independent risk factors for CL. Stratified analyses further demonstrated that younger males and females remained particularly vulnerable, underscoring the need for age‐ and gender‐sensitive prevention strategies. Importantly, the development of a clinically applicable risk scoring system represents a novel and pragmatic contribution of this study, offering a tool for early risk stratification, optimized surveillance and efficient allocation of limited healthcare resources (Salman et al. 1999; de Vries et al. 2015).

The association observed between marital status and CL risk should be interpreted with caution, as marital status is closely correlated with age in most populations. Younger individuals are more likely to be unmarried, and the higher infection rates observed among unmarried participants may therefore partially reflect the underlying age distribution rather than an independent causal relationship. Although multivariate analyses attempted to control for this potential confounding, residual confounding cannot be entirely excluded. Similar observations have been reported in other epidemiological studies where demographic variables such as marital status serve as proxies for behavioural exposure patterns rather than direct biological risk factors. Future studies using longitudinal designs and more detailed behavioural data would be helpful to disentangle the independent effects of age, marital status and lifestyle‐related exposure factors on CL transmission.

### 4.1 Study Limitations

This study has several limitations that should be acknowledged. First, the cross‐sectional study design limits the ability to establish causal relationships between identified risk factors and CL occurrence, as exposure and outcome were measured at the same time point. Second, although efforts were made to recruit participants from multiple healthcare facilities and community settings, the sampling approach may not fully capture all cases in the community, particularly individuals who did not seek medical care, potentially introducing selection bias. Third, important environmental and ecological variables such as housing conditions, proximity to vector breeding sites, vegetation cover and animal reservoirs were not measured, which may influence disease transmission dynamics. In addition, although laboratory confirmation was performed for diagnosis, species‐level molecular characterization of *Leishmania* parasites was not systematically conducted for all samples, which limits insights into species‐specific transmission patterns. These limitations should be considered when interpreting the findings, and future studies incorporating longitudinal designs, environmental assessments and molecular characterization would provide a more comprehensive understanding of CL epidemiology in the region.

## 5 Conclusion

This study provides a detailed epidemiological landscape of CL in District Bajaur, identifies high‐risk populations and offers actionable insights for public health planning. Targeted interventions focusing on younger males, high‐prevalence tehsils and peak transmission seasons are likely to be the most effective in reducing the burden of this neglected tropical disease.

## Author Contributions

**Murad Ali Khan**: conceptualization, funding acquisition. **Jawad Ali**: methodology, investigation. **Zohaib Ali**: visualization, validation. **Abdur Rehman**: software, data curation. **Muhammad Shoib**: supervision. **Sadaf Fayaz**: investigation. **Rifat Ullah Khan**: writing – review and editing, writing – original draft. **Shabana Naz**: writing – original draft, writing – review and editing. **Naseer Khan Momand**: writing – review and editing, writing – original draft. **Antonella Perillo**: writing – review and editing, writing – original draft. **Ibrahim A. Alhidary**: funding acquisition.

## Ethics Statement

The study was approved by the Ethical Committee of the Faculty of Animal Husbandry & Veterinary Sciences, The University of Agriculture, Peshawar, Pakistan (Approval No. 12/CVS/2024).

## Conflicts of Interest

The authors declare no conflicts of interest.

## Data Availability

The relevant data are provided in the paper. The data of the current experiment can be obtained from the corresponding author upon request.

## References

[vms371006-bib-0001] Abadías‐Granado, I. , A. Diago , P. A. Cerro , A. M. Palma‐Ruiz , and Y. J. Gilaberte . 2021. “Cutaneous and Mucocutaneous Leishmaniasis.” Actas Dermo‐Sifiliográficas (English Edition) 112: 601–618. 10.1016/j.adengl.2020.09.001. 34045157

[vms371006-bib-0002] Afghan, A. K. , M. Kassi , P. M. Kasi , A. Ayub , N. Kakar , and S. M. Marri . 2011. “Clinical Manifestations and Distribution of Cutaneous Leishmaniasis in Pakistan.” Journal of Tropical Medicine 2011: 359145. 10.1155/2011/359145. 22174721 PMC3235881

[vms371006-bib-0003] Bamorovat, M. , I. Sharifi , S. Agha Kuchak Afshari , and P. Ghasemi Nejad Almani . 2023. “Mutual Role of Patients and the Healthcare System in the Control of Cutaneous Leishmaniasis.” Transboundary and Emerging Diseases 2023: 7814940. 10.1111/tbed.14874. 40303822 PMC12016944

[vms371006-bib-0004] Carvalho, A. M. , C. F. Amorim , J. L. Barbosa , A. S. Lago , and E. M. Carvalho . 2015. “Age Modifies the Immunologic Response and Clinical Presentation of American Tegumentary Leishmaniasis.” American Journal of Tropical Medicine and Hygiene 92: 1173. 10.4269/ajtmh.14-0747. 25918209 PMC4458822

[vms371006-bib-0005] de Vries, H. J. , S. H. Reedijk , and H. D. Schallig . 2015. “Cutaneous Leishmaniasis: Recent Developments in Diagnosis and Management.” American Journal of Clinical Dermatology 16: 99–109. 10.1007/s40257-014-0100-0. 25687688 PMC4363483

[vms371006-bib-0006] de Vries, H. J. , and H. D. Schallig . 2022. “Cutaneous Leishmaniasis: A 2022 Updated Narrative Review Into Diagnosis and Management Developments.” American Journal of Clinical Dermatology 23: 823–840. 10.1007/s40257-022-00701-0. 36103050 PMC9472198

[vms371006-bib-0007] Hussain, M. , S. Munir , M. A. Jamal , S. Ayaz , M. Akhoundi , and K. Mohamed . 2017. “Epidemic Outbreak of Anthroponotic Cutaneous Leishmaniasis in Kohat District, Khyber Pakhtunkhwa, Pakistan.” Acta Tropica 172: 147–155. 10.1016/j.actatropica.2017.02.001. 28476600

[vms371006-bib-0008] Hussain, M. , S. Munir , T. A. Khan , et al. 2018. “Epidemiology of Cutaneous Leishmaniasis Outbreak, Waziristan, Pakistan.” Emerging Infectious Diseases 24: 159. 10.3201/eid2401.161493. 29260674 PMC5749458

[vms371006-bib-0009] Khan, K. , N. H. Khan , and S. Wahid . 2021. “Systematic Review of Leishmaniasis in Pakistan: Evaluating Spatial Distribution and Risk Factors.” Journal of Parasitology 107: 630–638. 10.1645/21-38. 34358311

[vms371006-bib-0010] Khattak, F. A. , N. Khan Wazir , H. Zia , et al. 2025. “Quality of Life in Pediatric Cutaneous Leishmaniasis: The Role of Lesion Type, Location, and Treatment.” INQUIRY: The Journal of Health Care Organization, Provision, and Financing 62: 00469580251344057. 10.1177/00469580251344057. PMC12368411 40509689

[vms371006-bib-0011] Lu, C. , Z. Ullah , K. Khan , S. U. Shah , M. Jamal , and N. H. Khan . 2024. “Environmental and Socio‐Demographic Factors Associated With Cutaneous Leishmaniasis in District Khyber, Pakistan; Alarming Spread of the Disease to New Foci.” Heliyon 10: e04812. 10.1016/j.heliyon.2024.e04812. PMC11053183 38681643

[vms371006-bib-0012] Machado‐Coelho, G. L. , W. T. Caiaffa , O. Genaro , P. A. Magalhaes , and W. Mayrink . 2005. “Risk Factors for Mucosal Manifestation of American Cutaneous Leishmaniasis.” Transactions of the Royal Society of Tropical Medicine and Hygiene 99: 55–61. 10.1016/j.trstmh.2004.06.012. 15550262

[vms371006-bib-0013] Merdekios, B. , M. Shewangizaw , A. Sappo , et al. 2025. “Unveiling the Hidden Burden: Exploring the Psychosocial Impact of Cutaneous Leishmaniasis Lesions and Scars in Southern Ethiopia.” PLoS ONE 20: e0317576. 10.1371/journal.pone.0317576. 39908238 PMC11798448

[vms371006-bib-0014] Mohammadbeigi, A. , S. Khazaei , H. Heidari , et al. 2021. “An Investigation of the Effects of Environmental and Ecologic Factors on Cutaneous Leishmaniasis in the Old World: A Systematic Review Study.” Reviews on Environmental Health 36: 117–128. 10.1515/reveh-2020-0073. 32892182

[vms371006-bib-0015] Naz, S. , M. Nalcaci , O. Hayat , et al. 2024. “Genetic Diversity and Epidemiological Insights Into Cutaneous Leishmaniasis in Pakistan: A Comprehensive Study on Clinical Manifestations and Molecular Characterization of *Leishmania* Species.” Parasitology Research 123: 320. 10.1007/s00436-024-07920-7. 39254766

[vms371006-bib-0016] Polidano, K. , B. Wenning , A. Ruiz‐Cadavid , et al. 2022. “Community‐Based Interventions for the Prevention and Control of Cutaneous Leishmaniasis: A Systematic Review.” Social Sciences 11: 490. 10.3390/socsci11100490.

[vms371006-bib-0017] Rafique, A. , S. S. Sani , S. Sultana , T. Sultana , A. Ashraf , and M. S. Mahmood . 2023. “Cutaneous Leishmaniasis.” In Leishmania Parasites–Epidemiology, Immunopathology and Hosts. IntechOpen. 10.5772/intechopen.113705.

[vms371006-bib-0018] Rostami, M. N. , A. Saghafipour , and E. Vesali . 2013. “A Newly Emerged Cutaneous Leishmaniasis Focus in Central Iran.” International Journal of Infectious Diseases 17: e1198–e1206. 10.1016/j.ijid.2013.07.012. 24011629

[vms371006-bib-0019] Salman, S. M. , N. G. Rubeiz , and A. G. Kibbi . 1999. “Cutaneous Leishmaniasis: Clinical Features and Diagnosis.” Clinics in Dermatology 17: 291–296. 10.1016/S0738-081X(99)00040-2. 10384868

[vms371006-bib-0020] Shaikh, F. M. , S. Raja , A. Ali , et al. 2025. “Epidemiology of Cutaneous Leishmaniasis in Karachi, Pakistan.” JAAD International 21: 9–15. 10.1016/j.jaad.2024.10.012. 40453505 PMC12123336

[vms371006-bib-0021] Suprien, C. , P. N. Rocha , M. Teixeira , et al. 2020. “Clinical Presentation and Response to Therapy in Children With Cutaneous Leishmaniasis.” American Journal of Tropical Medicine and Hygiene 102: 777. 10.4269/ajtmh.19-0640. 32043440 PMC7124913

[vms371006-bib-0022] Valero, N. N. H. , and M. Uriarte . 2020. “Environmental and Socioeconomic Risk Factors Associated With Visceral and Cutaneous Leishmaniasis: A Systematic Review.” Parasitology Research 119: 365–384. 10.1007/s00436-019-06576-w. 31897789

